# The Neutrophil-to-Albumin Ratio (NAR) Reflects the Severity of the Post-CABG Inflammatory Response and Is Associated with a Pre-Existing Pro-Inflammatory Monocyte Profile

**DOI:** 10.3390/life15121790

**Published:** 2025-11-21

**Authors:** Mikhail A. Popov, Siarhei A. Dabravolski, Vladislav V. Dontsov, Sergei A. Vzvarov, Evgeniy G. Agafonov, Dmitriy I. Zybin, Alexandra K. Kharabet, Olga V. Radchenkova, Dmitriy R. Saveliev, Victoria P. Pronina, Svetlana S. Verkhova, Nikita G. Nikiforov, Yegor S. Chegodaev, Alexander D. Zhuravlev, Daiana B. Erdyneeva, Yegor E. Yegorov, Elena E. Sigaleva, Milena I. Koloteva, Ekaterina V. Silina, Victor A. Stupin, Alexander V. Ivanov, Dmitriy V. Shumakov

**Affiliations:** 1 M.F. Vladimirsky Moscow Regional Clinical Research Institute, Schepkina Street 61/2, Moscow 129110, Russia; popovcardio88@mail.ru (M.A.P.); vvdontsov@yandex.ru (V.V.D.); sergejvzvarov@gmail.com (S.A.V.); agafonov.cardiacsurger@mail.ru (E.G.A.); poison1983@inbox.ru (D.I.Z.); akharabet@mail.ru (A.K.K.); morgunovaolya@yandex.ru (O.V.R.); dima_saveliev14072000@mail.ru (D.R.S.); vpronina85@gmail.com (V.P.P.); sdvtranspl@rambler.ru (D.V.S.); 2 Institute of General Pathology and Pathophysiology, Baltiyskaya Street 8, Moscow 125315, Russia; verxova.svetlana@gmail.com (S.S.V.); nikiforov.mipt@googlemail.com (N.G.N.); egozavr-ch@mail.ru (Y.S.C.); zhuravel17@yandex.ru (A.D.Z.); daya-na@mail.ru (D.B.E.); yegorov58@gmail.com (Y.E.Y.); sigaleva@mail.ru (E.E.S.); milenakoloteva@mail.ru (M.I.K.); ivanov_av82@mail.ru (A.V.I.); 3 Department of Biotechnology Engineering, Braude Academic College of Engineering, Snunit 51, P.O. Box 78, Karmiel 2161002, Israel; 4 I. M. Sechenov First Moscow State Medical University (Sechenov University), Moscow 119991, Russia; silinaekaterina@mail.ru; 5 Pirogov Russian National Research Medical University, Moscow 117997, Russia; stvictor@bk.ru

**Keywords:** coronary artery disease, coronary artery bypass grafting, inflammation, monocyte subsets, neutrophil-to-albumin ratio, biomarker, perioperative care, cardiac surgery

## Abstract

Background: The systemic inflammatory response to coronary artery bypass grafting (CABG) is highly variable and a key driver of complications. We hypothesised that a pre-existing pro-inflammatory immune state, characterised by a skewed monocyte profile, ‘primes’ patients for an exaggerated response. This pilot prospective study aimed to test this hypothesis and to evaluate the Neutrophil-to-Albumin Ratio (NAR) as an integrated biomarker of this response, comparing it against the established Neutrophil-to-Lymphocyte Ratio (NLR). Methods: In this pilot prospective, single-centre pilot study, we enrolled 34 patients with multivessel coronary artery disease (CAD) scheduled for off-pump CABG and 20 control subjects. Preoperatively, peripheral blood monocyte subsets were quantified by flow cytometry. Neutrophil, lymphocyte, and albumin levels were measured before and after surgery to calculate NAR and NLR. Multivariable linear regression was used to test for independent predictors of the inflammatory response. Results: Preoperatively, CAD patients exhibited a reduced percentage of the classical monocyte subpopulation (*p* < 0.001), with a skew toward intermediate and non-classical subpopulations. Postoperatively, both NAR and NLR increased significantly (*p* < 0.001) and performed comparably in discriminating the postoperative state (AUC: 0.89 vs. 0.86, *p* > 0.05). Critically, in multivariable linear regression analysis, the preoperative percentage of classical monocytes remained a significant and independent predictor of the magnitude of the postoperative NAR surge (β = −0.028, *p* = 0.007), after adjusting for clinical confounders including atherosclerotic burden. Conclusion: A patient’s preoperative immune profile, specifically the degree of monocyte skew, is an independent predictor of the acute inflammatory response to CABG. This finding supports a ‘priming’ mechanism in high-risk patients. While NAR and NLR perform similarly as monitoring tools, the independent link between the underlying immunology and the postoperative outcome suggests that combining preoperative immunophenotyping with simple biomarker monitoring could offer a powerful new strategy for personalised risk stratification in cardiac surgery.

## 1. Introduction

Cardiovascular diseases (CVDs), particularly coronary artery disease (CAD), remain the leading cause of mortality and morbidity worldwide, placing a substantial burden on healthcare systems globally [1]. Despite significant advances in prevention and treatment, the prevalence of CAD continues to rise, driven by an ageing population and the increasing incidence of risk factors such as diabetes, obesity, and hypertension [1,2]. For patients with advanced, multivessel CAD, coronary artery bypass grafting (CABG) remains a cornerstone of therapy, effectively improving survival and quality of life. However, a key clinical challenge is that the clinical benefits of CABG can be offset by a significant systemic inflammatory response to the surgical trauma, which is a major determinant of postoperative complications and recovery [3,4].

The pathophysiology of atherosclerosis is now understood to be intrinsically linked to chronic, low-grade inflammation [5,6], which may prime patients for this exaggerated response. Within this process, circulating monocytes play a central role. These cells are not a homogenous population but comprise distinct subsets—classical (CD14++CD16^−^), intermediate (CD14++CD16^+^), and non-classical (CD14^+^CD16^++^)—each with unique functional properties [7]. A growing body of evidence indicates that the balance between these subsets is disrupted in CAD, with a characteristic shift away from homeostatic classical monocytes towards pro-inflammatory intermediate and non-classical populations. This skewing is associated with increased atherosclerotic plaque burden and a higher risk of adverse cardiovascular events, suggesting that the preoperative immune profile of a patient is not a passive background but an active determinant of their underlying disease state a patient’s preoperative immune profile is an active component of their disease state [8,9].

Major surgery, such as CABG, imposes an acute “second hit” of inflammatory stress upon this chronically activated immune system. The resulting systemic inflammatory response syndrome (SIRS) is characterised by a surge in pro-inflammatory cytokines, activation of leucocytes, and a profound metabolic and catabolic shift [3,4,10]. The intensity of this response is highly variable among individuals and is closely linked to postoperative outcomes, including organ dysfunction, infection, and prolonged hospital stays. Consequently, there is a pressing clinical need for accessible and reliable biomarkers that can both predict and monitor this perioperative inflammatory trajectory.

While sophisticated markers like Interleukin-6 (IL-6) provide valuable mechanistic insight, their routine clinical use is often impractical. This has led to the investigation of simpler, integrated biomarkers derived from routine laboratory tests. Ratios such as the Neutrophil-to-Lymphocyte Ratio (NLR) have shown promise, but as they primarily reflect leucocyte balance, they may not fully capture the metabolic dimension of surgical stress [11,12,13]. The Neutrophil-to-Albumin Ratio (NAR) is an emerging biomarker that elegantly combines a more comprehensive assessment by combining two key aspects of the postoperative response: the neutrophil count, representing the acute cellular inflammatory surge, and the serum albumin level, a robust indicator of the negative acute-phase response, catabolism, and nutritional status [14,15,16].

However, it remains unclear whether a patient’s preoperative immune signature is linked to the magnitude of their subsequent inflammatory response to surgery. Establishing such a link would be a critical step towards personalised perioperative risk stratification. Therefore, the primary aim of this pilot study was twofold: first, to characterise the preoperative monocyte subpopulation profile in patients with severe CAD and, second, to investigate whether this baseline immune state is associated with the postoperative inflammatory response, as quantified by NAR. We hypothesised that a preoperative pro-inflammatory monocyte skew would be associated with a more pronounced postoperative inflammatory surge, and that NAR would serve as an effective biomarker to track this dynamic process. We also sought to compare the performance of NAR against the established NLR.

## 2. Materials and Methods

### 2.1. Study Design and Ethical Approval

This was a prospective, single-centre, observational pilot study conducted at the Vladimirsky Moscow Regional Clinical Research Institute (MONIKI), Moscow, Russia, between September 2024 and March 2025.

The study was performed in strict accordance with the principles of the Declaration of Helsinki (2013 revision) and conformed to Good Clinical Practice (GCP) guidelines. The study protocol was reviewed and approved by the Local Ethics Committee of the Moscow Regional Research and Clinical Institute (MONIKI) (Protocol No. 8, dated 17 May 2020). All participants provided written informed consent prior to their inclusion in the study and any study-related procedures.

### 2.2. Patient and Control Cohorts

The patient cohort consisted of 34 individuals with stable, multivessel coronary artery disease (CAD) scheduled for elective off-pump coronary artery bypass grafting (CABG). The control group comprised 20 individuals who underwent coronary angiography for clinical indications (e.g., atypical chest pain) but were found to have no evidence of significant coronary atherosclerosis.

Inclusion criteria for the patient group were: Age between 40 and 70 years; Diagnosis of stable multivessel CAD requiring elective off-pump CABG, defined as angiographic evidence of ≥70% stenosis in two or more major epicardial arteries.

Exclusion criteria for both groups were: Acute myocardial infarction within the preceding 30 days; Chronic heart failure of New York Heart Association (NYHA) functional class III–IV; Known active oncological or haematological disorders; Active infectious or chronic inflammatory diseases (e.g., rheumatoid arthritis); Requirement for emergency surgical intervention; Diagnosed non-ischaemic cardiomyopathies; Severe renal dysfunction (eGFR < 30 mL/min/1.73 m^2^) or severe hepatic dysfunction.

The severity of coronary atherosclerosis in the patient group was quantified using the Gensini score, calculated from the coronary angiograms by two independent cardiologists blinded to the patients’ immunological data. Baseline medication usage, including statins, ACE inhibitors, and beta-blockers, was recorded for all participants.

### 2.3. Data and Biological Sample Collection

For all participants, baseline demographic, clinical, and angiographic data were collected from medical records.

For the patient group, peripheral venous blood samples (35 mL into K_2_EDTA-containing tubes) were collected at two time points:

1. Preoperatively: On the morning of surgery, after an overnight fast.
2. Postoperatively: On the morning of postoperative day 2, to coincide with the typical peak of the systemic inflammatory response.

For the control group, a single fasting blood sample was collected at the time of recruitment.

### 2.4. Routine Clinical and Biochemical Analyses

Full blood counts, including absolute neutrophil and lymphocyte counts, were determined from K_2_EDTA whole blood using an automated haematology analyser (Sysmex XN-1000, Sysmex Corporation, Kobe, Japan).

Serum samples, obtained after centrifugation, were used for the measurement of biochemical parameters. Total protein, albumin, C-reactive protein (CRP), ferritin, and lipid profiles were measured on an automated biochemical analyser (Roche Cobas 8000, Roche Diagnostics, Basel, Switzerland) using standard commercially available reagent kits. Plasma levels of Interleukin-6 (IL-6) were measured using a commercially available high-sensitivity enzyme-linked immunosorbent assay (ELISA) kit (R&D Systems, Minneapolis, MN, USA).

### 2.5. Immunological Analyses

A 50 µL aliquot of the leucocyte fraction was stained with fluorescently conjugated monoclonal antibodies against CD14 (FITC conjugate) and CD16 (PB450-A conjugate) (Miltenyi Biotec, Bergisch Gladbach, Germany). Following a 15 min incubation at 4 °C in the dark, red blood cells were lysed, and the remaining cells were washed and resuspended in phosphate-buffered saline (PBS). Samples were analysed on a CytoFLEX V2-B3-R2 flow cytometer (Beckman Coulter, Brea, CA, USA). Monocytes were first gated based on their forward scatter (FSC-A) and side scatter (SSC-A) characteristics. Within this gate, subpopulations were identified as: classical (CD14++CD16^−^), intermediate (CD14++CD16^+^), and non-classical (CD14^+^CD16^++^).

Peripheral blood mononuclear cells (PBMCs) were isolated from preoperative whole blood samples by density gradient centrifugation over Ficoll-Paque PLUS (density 1.077 g/mL; Paneco, Russia). Primary monocytes were then purified from the PBMC fraction by positive selection using CD14 MicroBeads (Miltenyi Biotec) and magnetic-activated cell sorting (MACS) according to the manufacturer’s protocol. Purity and viability of isolated monocytes were confirmed to be >95%. Cells were resuspended in X-Vivo 15 serum-free medium (Lonza, Basel, Switzerland) and seeded into 24-well plates at a concentration of 1 × 10^6^ cells/mL. The cells were cultured at 37 °C in a humidified atmosphere with 5% CO_2_. Monocytes were either left unstimulated (basal condition) or stimulated with 1 µg/mL of lipopolysaccharide (LPS from *E. coli* O111:B4; Sigma-Aldrich, St. Louis, MO, USA) for 24 h.

After 24 h of incubation, culture supernatants were collected, centrifuged to remove cellular debris, and stored at −80 °C until analysis. Concentrations of (TNF-α), IL-1β, IL-6, IL-10, IL-8 (CXCL8), and monocyte chemoattractant protein-1 (CCL2), key cytokines (TNF-α, IL-1β, IL-6, IL-10, IL-8, and CCL2) were quantified using commercial ELISA kits (R&D Systems, Minneapolis, MN, USA) following the manufacturer’s instructions. Optical density was measured at 450 nm with wavelength correction using a CLARIOstar Plus Microplate Reader (BMG LABTECH, Ortenberg, Germany).

### 2.6. Calculation of Inflammatory Ratios

The Neutrophil-to-Lymphocyte Ratio (NLR) and the Neutrophil-to-Albumin Ratio (NAR) were calculated for each relevant time point using the following formulae:

NLR = Absolute Neutrophil Count (×10^9^/L)/Absolute Lymphocyte Count (×10^9^/L)

NAR = Absolute Neutrophil Count (×10^9^/L)/Serum Albumin Concentration (g/L)

### 2.7. Statistical Analysis

Statistical analyses were performed using IBM SPSS Statistics for Windows, Version 26.0 (IBM Corp., Armonk, NY, USA) and GraphPad Prism Version 9.0 (GraphPad Software, San Diego, CA, USA).

The normality of data distribution was assessed using the Shapiro–Wilk test and visual inspection of Q-Q plots. Normally distributed data are presented as mean ± standard deviation (SD), while non-normally distributed data are presented as median with interquartile range (IQR).

For comparisons between two independent groups, the independent samples Student’s *t*-test or the Mann–Whitney U test was used as appropriate. For comparisons of paired data (preoperative vs. postoperative), the paired samples *t*-test or the Wilcoxon signed-rank test was used. Correlations between variables were assessed using Pearson’s correlation coefficient (r) for normally distributed data or Spearman’s rank correlation coefficient (ρ) for non-normally distributed data.

Receiver operating characteristic (ROC) curve analysis was used to evaluate the ability of NAR and NLR to distinguish between different clinical states. The area under the curve (AUC), with its 95% confidence interval (CI), was calculated, and the AUCs for the two ratios were formally compared using the method of DeLong et al. [17]. The optimal cut-off value was determined using the Youden index.

To investigate the independent association between preoperative monocyte subsets and the postoperative inflammatory response, a multivariable linear regression analysis was performed. The change in NAR (ΔNAR = Postoperative NAR − Preoperative NAR) was used as the dependent variable. Independent variables included the percentage of classical monocytes and clinically relevant confounders such as age, BMI, Gensini score, and presence of diabetes mellitus.

A two-tailed *p*-value < 0.05 was considered statistically significant for all analyses.

## 3. Results

### 3.1. Baseline Clinical and Immunological Profile of Study Participants

The study included 34 patients with multivessel CAD scheduled for off-pump CABG and 20 control subjects without significant coronary atherosclerosis. Baseline clinical, demographic, and medication data are presented in Table 1.

The patient and control groups were well-matched for age, sex, and Body Mass Index (BMI). Use of key cardiovascular medications such as statins and ACE inhibitors was high in the patient group, as expected. As anticipated, patients with CAD had a significantly higher atherosclerotic burden, evidenced by a high mean Gensini score (86.3 ± 16.2), and a lower left ventricular ejection fraction (LVEF) compared to controls (55% vs. 64%, *p* < 0.001).

**Figure 1:**
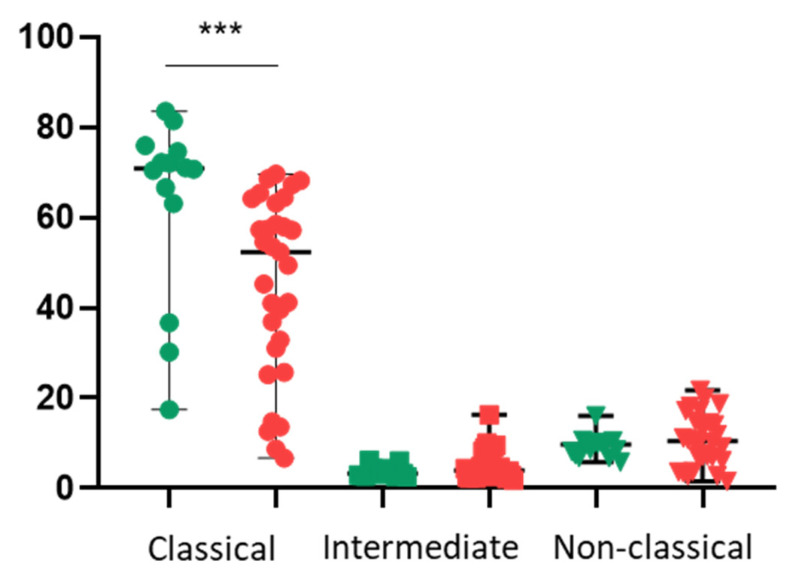
Monocyte subpopulation distribution in control subjects and patients with CAD. Percentage of classical (CD14++CD16^−^), intermediate (CD14++CD16^+^), and non-classical (CD14^+^CD16^++^) monocytes in the peripheral blood of control subjects (green) and preoperative patients with CAD (red). Statistical significance was determined using the Mann–Whitney U test. *** *p* < 0.001.

Critically, the baseline immune profile differed significantly between the groups. Patients with CAD exhibited a distinct monocyte subpopulation skew, characterised by a lower percentage of classical (CD14++CD16^−^) monocytes compared to the control group. Conversely, there was a trend towards higher percentages of intermediate and non-classical monocytes in the patient cohort (Figure 1).

### 3.2. Functional Profile of Circulating Monocytes

**Figure 2:**
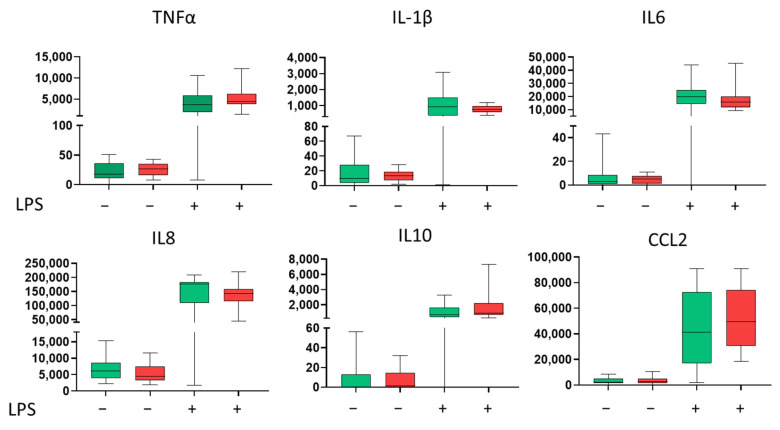
Basal and lipopolysaccharide (LPS)-stimulated cytokine secretion by primary monocytes. Concentration (pg/mL) in the 24 h culture supernatants of primary monocytes isolated from control subjects (green) (*n* = 20) and preoperative CAD patients (red) (*n* = 34). Cells were cultured under either basal (unstimulated) or LPS-stimulated (1 µg/mL) conditions. Green represents controls, and red—CAD patients. Statistical significance was calculated using the Mann–Whitney U test, as the data did not follow a normal distribution. The results are presented as box plots, where the centre line represents the median, the box boundaries indicate the first and third quartiles (Q1 and Q3), and the whiskers show the minimum and maximum values.

Primary monocytes were isolated and their cytokine secretion was assessed at baseline and after stimulation with lipopolysaccharide (LPS). Monocytes from CAD patients showed a tendency towards higher LPS-stimulated secretion of TNFα, IL-10, and CCL2, but the difference did not reach statistical significance. (Figure 2).

### 3.3. The Systemic Inflammatory Response to Off-Pump CABG

Surgical intervention induced a profound systemic inflammatory and catabolic response in the patient group. As shown in Table 2, there was a multi-fold increase in the plasma levels of inflammatory markers from the preoperative to the postoperative period. This was accompanied by a marked leucocytosis, driven primarily by a surge in neutrophils (5.9 to 10.7 × 10^9^/L, *p* < 0.001). This was accompanied by a marked leucocytosis, driven by a surge in neutrophils and a corresponding lymphopenia. Concurrently, there was evidence of significant catabolism and haemodilution, with a marked fall in serum albumin from 41.3 to 34.1 g/L (*p* < 0.001).

### 3.4. Performance of NAR and NLR as Biomarkers of the Postoperative Response

**Figure 3:**
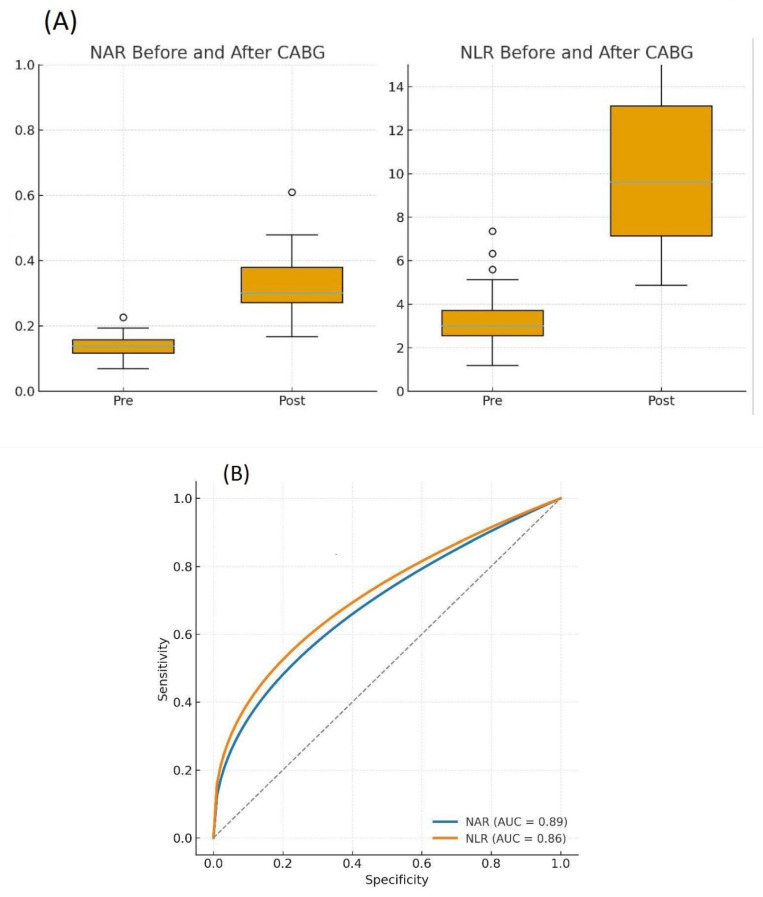
Dynamics and diagnostic performance of the Neutrophil-to-Albumin Ratio (NAR) and Neutrophil-to-Lymphocyte Ratio (NLR). (**A**) Paired plots showing the significant increase in NAR and NLR from the preoperative to the postoperative period in CAD patients (*n* = 34). Significance was determined by the Wilcoxon signed-rank test. *p* < 0.0001. (**B**) Receiver operating characteristic (ROC) curves comparing the ability of postoperative NAR (blue line) and NLR (orange line) to discriminate the postoperative from the preoperative state. The area under the curve (AUC) with 95% confidence intervals (CI) is shown for each ratio.

The Neutrophil-to-Albumin Ratio (NAR) was calculated to provide an integrated measure of the inflammatory-catabolic state. Both the Neutrophil-to-Albumin Ratio (NAR) and the Neutrophil-to-Lymphocyte Ratio (NLR) increased significantly in the postoperative period (*p* < 0.001 for both) (Figure 3A).

ROC curve analysis was performed to compare the ability of postoperative NAR and NLR to distinguish the postoperative state from the preoperative condition. Both ratios performed well, but NAR demonstrated a numerically higher area under the curve (AUC) than NLR (NAR AUC: 0.89, 95% CI 0.79–0.97 vs. NLR AUC: 0.86, 95% CI 0.75–0.95)). However, this difference was not statistically significant (*p* = 0.27) (Figure 3B and Table 3). An optimal NAR cut-off value of 0.21 provided a sensitivity of 82.4% and a specificity of 100% for identifying the postoperative state.

### 3.5. The Preoperative Immune State Is an Independent Predictor of the Postoperative Response

Finally, we investigated whether the preoperative immunological profile was associated with the magnitude of the postoperative inflammatory-catabolic response. We next sought to determine if the preoperative immune profile was associated with the magnitude of the postoperative response. A strong positive correlation was observed between the preoperative percentage of classical monocytes and the postoperative serum albumin level (r = 0.776, *p* = 0.001), suggesting that a more homeostatic preoperative immune profile was associated with better preservation of metabolic status after surgery.

**Figure 4:**
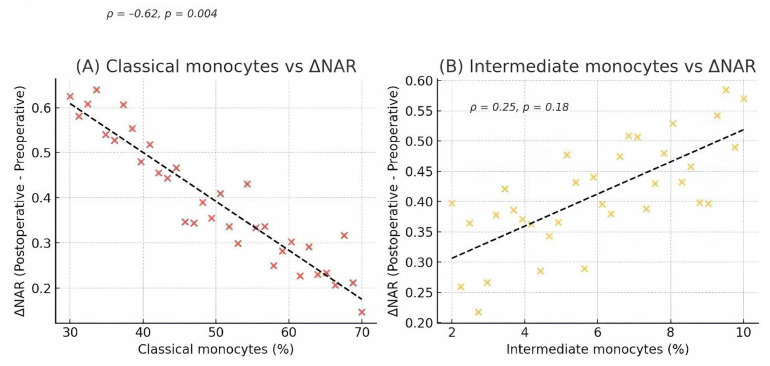
Correlation between preoperative monocyte subsets and the postoperative NAR response. Scatter plots showing the correlation between preoperative monocyte percentages and the change in the Neutrophil-to-Albumin Ratio (ΔNAR = Postoperative NAR − Preoperative NAR) in CAD patients (*n* = 34). (**A**) A significant negative correlation was observed between the percentage of classical monocytes and ΔNAR. (**B**) A positive, though non-significant, correlation was observed between the percentage of intermediate monocytes and ΔNAR. Each point represents an individual patient. The line represents the linear regression fit, with Spearman’s rank correlation coefficient (ρ) and *p*-value shown on each plot.

To directly test our primary hypothesis, we correlated baseline monocyte percentages with the change in NAR (ΔNAR). A significant inverse correlation was found between the percentage of classical monocytes and ΔNAR (ρ = −0.62, *p* = 0.004), indicating that a greater preoperative skew away from classical monocytes predicted a more severe subsequent inflammatory response (Figure 4).

Finally, to assess whether this association was independent of other clinical factors, we performed a multivariable linear regression analysis. After adjusting for age, BMI, Gensini score, and diabetes mellitus, the preoperative percentage of classical monocytes remained a significant, independent predictor of ΔNAR (β = −0.028, *p* = 0.004) (Table 4).

## 4. Discussion

In this prospective pilot study, we investigated the interplay between the preoperative immune state and the acute systemic inflammatory response to off-pump CABG surgery. Our findings present a cohesive “bench-to-bedside” narrative with three key conclusions. First, patients with severe coronary artery disease exhibit a distinct pro-inflammatory immunological signature at baseline, characterised by a significant skewing of monocyte subpopulations. Second, the simple NAR serves as an effective integrated biomarker for quantifying the postoperative inflammatory-catabolic response, performing comparably to the established NLR. Third, and most importantly, we establish a novel, independent link between these two observations: the degree of preoperative monocyte skewing is directly associated with the intensity of the postoperative inflammatory surge, as measured by NAR, even after accounting for traditional clinical risk factors.

### 4.1. The Pre-Activated Immune State in Coronary Artery Disease

Our initial finding confirms the well-established role of chronic inflammation in the pathophysiology of atherosclerosis [5]. The significant reduction in circulating classical monocytes in our CAD cohort aligns with a growing body of evidence implicating a shift towards pro-inflammatory intermediate and non-classical subsets in atherosclerotic plaque formation and destabilisation [18,19,20]. Our functional assays, while limited by statistical power, suggested a tendency for these monocytes to be ‘primed’ for a hyper-inflammatory response. This supports a “first hit” model, whereby the chronic inflammatory milieu of advanced atherosclerosis leads not only to a phenotypic shift in circulating monocytes but also to a state of heightened readiness to respond to a “second hit,” such as major surgery.

### 4.2. NAR as a Robust Marker of Surgical Stress

The postoperative period following major cardiac surgery is characterised by a complex systemic inflammatory response syndrome (SIRS), and monitoring this response is crucial for identifying patients at risk [21,22]. Our study validates NAR as a practical tool for this purpose. NAR uniquely integrates two critical components of the postoperative state: the neutrophilic inflammatory surge and the catabolic/negative acute-phase response reflected by hypoalbuminaemia, which is likely a composite of the inflammatory response and perioperative haemodilution. Our data showed that NAR’s ability to discriminate the postoperative state was excellent (AUC = 0.89) and was numerically, though not statistically, superior to the widely used NLR. This suggests that the inclusion of albumin, a direct marker of metabolic stress, may offer a more comprehensive assessment of the patient’s overall condition than leucocyte ratios alone. Similar results are confirmed by data from recent studies, where NAR has demonstrated prognostic significance in acute inflammatory conditions, including sepsis, ischaemic stroke, and pneumonia [23,24,25,26,27]. This underscores the universality of this index as a marker of the systemic inflammatory and catabolic response. In the context of cardiac surgery, where early detection of hyperinflammation is critical for preventing organ dysfunction, NAR could be incorporated into standard monitoring algorithms. Its advantage lies in the combination of high sensitivity and accessibility, which makes it a promising tool for risk stratification in routine practice, while the simplicity and low cost, derived from two of the most common laboratory tests, make NAR a universally applicable tool for perioperative monitoring.

### 4.3. The Novel Link: Preoperative Immunity as an Independent Predictor of Postoperative Response

A key finding of our study is the direct link between the preoperative immune landscape and the postoperative clinical trajectory. The strong correlation between a more homeostatic classical monocyte profile at baseline and better preservation of postoperative albumin levels provided the first hint of this connection.

Crucially, our primary hypothesis was confirmed by the multivariable analysis. The finding that the preoperative percentage of classical monocytes remained a significant, independent predictor of the postoperative surge in NAR—after adjusting for atherosclerotic burden (Gensini score), diabetes, and other confounders—is the cornerstone of this manuscript. This moves beyond simple association and suggests that the preoperative immune phenotype is not merely a surrogate for disease severity but is, in itself, a determinant of the patient’s capacity to handle the acute stress of surgery.

The proposed mechanism may involve the heightened sensitivity of monocytes, previously ‘primed’ by chronic inflammation, to a secondary stimulus (surgical stress), with subsequent hypersecretion of IL-6 and TNF-α. This is relevant because these cytokines enhance neutrophil mobilisation while simultaneously suppressing albumin synthesis in the liver, which is directly reflected by an increase in the NAR index. The data obtained are consistent with the concept of “immunobiological priming,” according to which chronic inflammation induces epigenetic reprogramming in innate immune cells, increasing their reactivity to subsequent exposures.

This concept is strongly supported by recent advances in our understanding of “trained immunity,” a mechanism described in studies of chronic atherosclerosis and diabetes where monocytes and macrophages retain an epigenetic memory of previous inflammatory insults [28,29,30,31,32,33]. This leads to enhanced production of IL-6, TNF-α, and other mediators upon re-stimulation, which likely explains the hyperinflammatory response after surgical stress observed in our patients. Thus, preoperative determination of the monocyte subpopulation profile could become not only a diagnostic but also a potentially therapeutic marker, allowing for the identification of patients who may benefit from targeted immunomodulation before surgery. This shifts the paradigm from simply reacting to postoperative inflammation to potentially predicting it based on a preoperative blood test.

### 4.4. Clinical Implications and Future Directions

Our results open prospects for a personalised approach to the management of patients undergoing CABG. Preoperative assessment of the monocyte profile combined with NAR monitoring could be used to predict the risk of a hyperinflammatory response and to adjust patient management tactics.

Specifically, patients with a pronounced skew away from classical monocytes could be candidates for:

- Intensification of anti-inflammatory therapy (e.g., optimising statin doses or using short courses of steroids/IL-6 blockers in the perioperative period);
- Nutritional and albumin support to minimise the catabolic response;
- Dynamic laboratory monitoring of NAR in the first 48–72 h after surgery.

In the future, multicentre studies are needed to refine the threshold values for NAR, determine the temporal dynamics of its changes, and integrate this indicator into existing prognostic scales, such as SCORE2.

### 4.5. Limitations

We must acknowledge several limitations inherent to this pilot exploratory study. First, the small sample size limits the statistical power of our analyses and the generalisability of our findings. The trends observed in the functional cytokine assays and the non-significant difference between NAR and NLR require validation in larger, multi-centre cohorts. Second, the observational design demonstrates association, not causation. While mechanistically plausible, we cannot definitively prove that the monocyte skew causes the exaggerated postoperative response. Third, our control group, while free of significant CAD, consisted of patients undergoing angiography for clinical reasons and may not represent a truly “healthy” population. Finally, although we adjusted for several key confounders, the possibility of residual confounding from unmeasured variables, such as specific medication dosages, remains.

## 5. Conclusions

This pilot study provides novel evidence that a preoperative pro-inflammatory monocyte profile is an independent predictor of the magnitude of the systemic inflammatory response following off-pump CABG. The simple and accessible Neutrophil-to-Albumin Ratio serves as a robust biomarker for quantifying this response. These findings, though preliminary, suggest that integrating the combination of preoperative immunoprofiling and simple laboratory biomarkers, such as NAR, could form the basis of personalised risk stratification in cardiac surgery. The inclusion of NAR in standard postoperative monitoring would allow for the early identification of patients with an excessive inflammatory response and potentially optimise therapy—for example, guiding doses of albumin infusions, nutritional support, or the use of targeted anti-inflammatory approaches. In the long term, it may be possible to evaluate the effectiveness of preoperative interventions aimed at modulating the monocyte profile (e.g., the use of statins, anti-cytokine strategies, or nutritional correction) to reduce postoperative risk. Further validation in larger prospective studies is warranted.

## Figures and Tables

**Table 1:** Baseline clinical, medication, and immunological characteristics of the control and patient groups.

Parameter	Control Group (*n* = 20)	Patient Preoperative (*n* = 34)	*p*-Value
Demographics & Clinical			
Age, years	60 [57.3–65.8]	59 [55–61]	0.023
Sex (Male), %	17 (85%)	26 (76.5%)	0.904
BMI (kg/m^2^)	28 [27.2–28.9]	28 [27.2–28.7]	0.692
Gensini score	−	86.3 ± 16.2	<0.001
LVEF, %	64 [59–65.8]	55 [50.8–57]	<0.001
Diabetes mellitus, %	9 (45%)	16 (47%)	0.884
Baseline Medications, %			
Statins	35%	91%	<0.001
ACE Inhibitors/ARBs	40%	85%	<0.001
Beta-blockers	45%	88%	<0.001
Aspirin	50%	94%	<0.001
Immunological Profile			
Classical monocytes, %	62.3 ± 19.8	45.3 ± 19.7	0.009
Intermediate monocytes, %	3.3 [2.8–4.4]	3.9 [2.9–6.1]	0.331
Non-classical monocytes, %	9.3 ± 2.4	10.5 ± 5.6	0.304

**Table 2:** Preoperative and postoperative laboratory parameters in the patient group (*n* = 34).

Parameter	Patient Preoperative	Patient Postoperative	*p*-Value
Inflammatory Markers			
CRP, mg/L	4.4 ± 2.2	26.7 ± 9.1	<0.001
IL-6, pg/mL	4.3 ± 1.7	21.1 ± 8.1	<0.001
Haematology			
WBC, ×10^9^/L	6.4 ± 1.3	20.0 [15.5–29.8]	<0.001
Neutrophils, ×10^9^/L	5.9 ± 1.5	10.7 [8.2–13.9]	<0.001
Lymphocytes, ×10^9^/L	1.9 ± 0.6	1.1 ± 0.4	<0.001
Biochemistry			
Albumin, g/L	41.3 ± 2.6	34.1 ± 4.9	<0.001

**Table 3:** Diagnostic performance of NAR and NLR in discriminating between preoperative and postoperative states.

Biomarker	AUC (95% CI)	Optimal Cut-Off	Sensitivity (%)	Specificity (%)
NAR	0.89 (0.79–0.97)	0.21	82.4	100
NLR	0.86 (0.75–0.95)	3.8	79.4	94.1

**Table 4:** Multivariable linear regression analysis for predictors of the change in NAR (ΔNAR).

Variable	Univariable β (95% CI)	*p*-Value	Multivariable β (95% CI)	*p*-Value
Classical Monocytes (%)	−0.031(−0.052; −0.010)	0.005	−0.028(−0.049; −0.009)	0.007
Age, years	0.004(−0.003; 0.011)	0.262	0.003(−0.004; 0.010)	0.351
BMI, kg/m^2^	0.012(−0.004; 0.028)	0.141	0.010(−0.005; 0.026)	0.184
Gensini Score	0.001(−0.001; 0.004)	0.292	0.001(−0.002; 0.003)	0.421
Diabetes Mellitus (Yes/No)	0.041(−0.018; 0.099)	0.174	0.037(−0.024; 0.088)	0.223

## Data Availability

Data are available on reasonable request. The data underlying this article cannot be shared publicly to protect the privacy of individuals who participated in this study. The anonymised data may be shared on reasonable request to the corresponding author.

## References

[1] Vos T., Lim S.S., Abbafati C., Abbas K.M., Abbasi M., Abbasifard M., Abbasi-Kangevari M., Abbastabar H., Abd-Allah F., Abdelalim A. (2020). Global Burden of 369 Diseases and Injuries in 204 Countries and Territories, 1990–2019: A Systematic Analysis for the Global Burden of Disease Study 2019. Lancet.

[2] Khan S.S., Breathett K., Braun L.T., Chow S.L., Gupta D.K., Lekavich C., Lloyd-Jones D.M., Ndumele C.E., Rodriguez C.J., Allen L.A. (2025). Risk-Based Primary Prevention of Heart Failure: A Scientific Statement From the American Heart Association. Circulation.

[3] El-Diasty M.M., Rodríguez J., Pérez L., Souaf S., Eiras S., Fernández A.L. (2024). Compartmentalization of the Inflammatory Response in the Pericardial Cavity in Patients Undergoing Cardiac Surgery. Int. J. Mol. Sci..

[4] Viikinkoski E., Aittokallio J., Lehto J., Ollila H., Relander A., Vasankari T., Jalkanen J., Gunn J., Jalkanen S., Airaksinen J. (2024). Prolonged Systemic Inflammatory Response Syndrome After Cardiac Surgery. J. Cardiothorac. Vasc. Anesth..

[5] Ait-Oufella H., Libby P. (2024). Inflammation and Atherosclerosis: Prospects for Clinical Trials. Arterioscler. Thromb. Vasc. Biol..

[6] Libby P. (2024). Inflammation and the Pathogenesis of Atherosclerosis. Vasc. Pharmacol..

[7] Ziegler-Heitbrock L., Ancuta P., Crowe S., Dalod M., Grau V., Hart D.N., Leenen P.J.M., Liu Y.-J., MacPherson G., Randolph G.J. (2010). Nomenclature of Monocytes and Dendritic Cells in Blood. Blood.

[8] Moroni F., Ammirati E., Norata G.D., Magnoni M., Camici P.G. (2019). The Role of Monocytes and Macrophages in Human Atherosclerosis, Plaque Neoangiogenesis, and Atherothrombosis. Mediat. Inflamm..

[9] Hristov M., Weber C. (2025). Monocyte Subsets in Cardiovascular Disease: A Biomarker Perspective. Thromb. Haemost..

[10] Zhao D., Yang R., Liu S., Ge D., Su X. (2023). Study on the Characteristics of Early Cytokine Storm Response to Cardiac Surgery. J. Interferon Cytokine Res..

[11] Tzikos G., Alexiou I., Tsagkaropoulos S., Menni A.-E., Chatziantoniou G., Doutsini S., Papavramidis T., Grosomanidis V., Stavrou G., Kotzampassi K. (2023). Neutrophil-to-Lymphocyte Ratio and Platelet-to-Lymphocyte Ratio as Predictive Factors for Mortality and Length of Hospital Stay after Cardiac Surgery. J. Pers. Med..

[12] Lim H.A., Kang J.K., Kim H.W., Song H., Lim J.Y. (2023). The Neutrophil-to-Lymphocyte Ratio as a Predictor of Postoperative Outcomes in Patients Undergoing Coronary Artery Bypass Grafting. J. Chest Surg..

[13] Bae M.I., Shim J.-K., Song J.W., Ko S.H., Choi Y.S., Kwak Y.-L. (2023). Predictive Value of the Changes in Neutrophil-Lymphocyte Ratio for Outcomes After Off-Pump Coronary Surgery. J. Inflamm. Res..

[14] Zhang X., Lin L., Peng Y., Li S., Huang X., Chen L., Lin Y. (2025). Neutrophil Percentage to Albumin Ratio Is Associated with In-Hospital Mortality in Patients with Acute Type A Aortic Dissection. J. Clin. Hypertens..

[15] Lei L., Liang Y., Chen J., Cui T., Fang J., Fei L., Lin W., Tang C., Jiang S., Wang X. (2025). Preoperative Neutrophil Percentage-to-Albumin Ratio as a Postoperative AKI Predictor in Non-Cardiac Surgery: A Retrospective Cohort Secondary Analysis. Sci. Rep..

[16] Li J., Yang M., Zhang X., Huang R., Zhang Y., Fan K. (2025). Neutrophil to Albumin Ratio Predicts Cardiovascular and All Cause Mortality in CVD Patients with Abnormal Glucose Metabolism. Sci. Rep..

[17] DeLong E.R., DeLong D.M., Clarke-Pearson D.L. (1988). Comparing the Areas under Two or More Correlated Receiver Operating Characteristic Curves: A Nonparametric Approach. Biometrics.

[18] Ruder A.V., Wetzels S.M.W., Temmerman L., Biessen E.A.L., Goossens P. (2023). Monocyte Heterogeneity in Cardiovascular Disease. Cardiovasc. Res..

[19] Czepluch F.S., Kuschicke H., Dellas C., Riggert J., Hasenfuss G., Schäfer K. (2014). Increased Proatherogenic Monocyte–Platelet Cross-talk in Monocyte Subpopulations of Patients with Stable Coronary Artery Disease. J. Intern. Med..

[20] Wildgruber M., Aschenbrenner T., Wendorff H., Czubba M., Glinzer A., Haller B., Schiemann M., Zimmermann A., Berger H., Eckstein H.-H. (2016). The “Intermediate” CD14++CD16+ Monocyte Subset Increases in Severe Peripheral Artery Disease in Humans. Sci. Rep..

[21] Thang N.V.V., Luyen L.T., Vi N.T.T., Hai P.D. (2025). Neutrophil-to-Lymphocyte-to-Albumin Ratio as a Prognostic Marker for Mortality in Sepsis and Septic Shock in Vietnam. Acute Crit. Care.

[22] Huang Z., Fu Z., Huang W., Huang K. (2020). Prognostic Value of Neutrophil-to-Lymphocyte Ratio in Sepsis: A Meta-Analysis. Am. J. Emerg. Med..

[23] Wan J., Liu Y., Yuan X., Fan S., Xiao Y., Fang F., Zhang Y. (2025). Neutrophil-to-Albumin Ratio Predicts Stroke-Associated Pneumonia in Patients with Intracerebral Hemorrhage. J. Inflamm. Res..

[24] Ari M., Haykir Solay A., Ozdemir T., Yildiz M., Mentes O., Tuten O.F., Tetik Manav H., Celik D., Doganci M., Eraslan Doganay G. (2025). Neutrophil Percentage-to-Albumin Ratio as a Prognostic Marker in Pneumonia Patients Aged 80 and Above in Intensive Care. J. Clin. Med..

[25] Lv X.-N., Shen Y.-Q., Li Z.-Q., Deng L., Wang Z.-J., Cheng J., Hu X., Pu M.-J., Yang W.-S., Xie P. (2023). Neutrophil Percentage to Albumin Ratio Is Associated with Stroke-Associated Pneumonia and Poor Outcome in Patients with Spontaneous Intracerebral Hemorrhage. Front. Immunol..

[26] Gong Y., Li D., Cheng B., Ying B., Wang B. (2020). Increased Neutrophil Percentage-to-Albumin Ratio Is Associated with All-Cause Mortality in Patients with Severe Sepsis or Septic Shock. Epidemiol. Infect..

[27] Zhang H., Wu T., Tian X., Lyu P., Wang J., Cao Y. (2021). High Neutrophil Percentage-To-Albumin Ratio Can Predict Occurrence of Stroke-Associated Infection. Front. Neurol..

[28] Dagenais A., Villalba-Guerrero C., Olivier M. (2023). Trained Immunity: A “New” Weapon in the Fight against Infectious Diseases. Front. Immunol..

[29] Mohammadnezhad L., Shekarkar Azgomi M., La Manna M.P., Sireci G., Rizzo C., Badami G.D., Tamburini B., Dieli F., Guggino G., Caccamo N. (2022). Metabolic Reprogramming of Innate Immune Cells as a Possible Source of New Therapeutic Approaches in Autoimmunity. Cells.

[30] Schlüter T., Van Elsas Y., Priem B., Ziogas A., Netea M.G. (2025). Trained Immunity: Induction of an Inflammatory Memory in Disease. Cell Res..

[31] Choudhury R.P., Edgar L., Rydén M., Fisher E.A. (2021). Diabetes and Metabolic Drivers of Trained Immunity: New Therapeutic Targets Beyond Glucose. Arterioscler. Thromb. Vasc. Biol..

[32] Edgar L., Akbar N., Braithwaite A.T., Krausgruber T., Gallart-Ayala H., Bailey J., Corbin A.L., Khoyratty T.E., Chai J.T., Alkhalil M. (2021). Hyperglycemia Induces Trained Immunity in Macrophages and Their Precursors and Promotes Atherosclerosis. Circulation.

[33] Van Tuijl J., Van Heck J.I.P., Bahrar H., Broeders W., Wijma J., Ten Have Y.M., Giera M., Zweers-van Essen H., Rodwell L., Joosten L.A.B. (2024). Single High-Fat Challenge and Trained Innate Immunity: A Randomized Controlled Cross-over Trial. iScience.

